# Prognostic value of Naples Prognostic Score in locally advanced cervical cancer patients undergoing concurrent chemoradiotherapy

**DOI:** 10.17305/bb.2024.10989

**Published:** 2024-08-15

**Authors:** Xiaojun Zhang, Mengxuan Gu, Jiahao Zhu, Ruike Gu, Bo Yang, Shengjun Ji, Yutian Zhao, Ke Gu

**Affiliations:** 1 Department of Radiotherapy and Oncology, The Affiliated Hospital of Jiangnan University, Wuxi, China; 2 Wuxi School of Medicine, Jiangnan University, Wuxi, China; 3 Department of Rehabilitation Medical, Suzhou Rehabilitation Hospital (Suzhou Municipal Hospital Rehabilitation Medical Center), Suzhou, China; 4 Department of Radiotherapy and Oncology, The Affiliated Suzhou Hospital of Nanjing Medical University, Gusu School, Nanjing Medical University, Suzhou, China

**Keywords:** Naples Prognostic Score (NPS), locally advanced cervical cancer (LACC), immunonutritional indicator, chemoradiotherapy, prognosis

## Abstract

This study aimed to investigate the prognostic value of the Naples Prognostic Score (NPS) in patients with locally advanced cervical cancer (LACC) who received curative concurrent chemoradiotherapy (CCRT). Clinicopathological data from 213 (training set) and 106 (validation set) LACC cases undergoing CCRT were retrospectively analyzed. The receiver operating characteristic curve (ROC) was used to compare the predictive ability of NPS and other indicators for survival. Cox proportional hazard regression was conducted for overall survival (OS) and progression-free survival (PFS). A prediction model using a nomogram was developed with independent prognostic factors in the training set and validated in the validation set. The 5-year OS for the NPS ═ 1, 2, and 3 groups was 56.8%, 45.4%, and 28.9% (*P* < 0.001), and the 5-year PFS for the NPS ═ 1, 2, and 3 groups was 44.9%, 36.7%, and 28.4% (*P* ═ 0.001), respectively. NPS showed better predictive ability for OS and PFS compared to other indicators. Multivariate regression analysis identified NPS as an independent prognostic factor for OS (*P* < 0.001) and PFS (*P* < 0.001). A predictive nomogram based on NPS was established and validated. The C-indices of the nomogram in the training set were 0.722 for OS and 0.683 for PFS, while in the validation set, the C-indices were 0.731 for OS and 0.693 for PFS. This study confirmed that preoperative NPS could serve as a useful independent prognostic factor in LACC patients treated with CCRT.

## Introduction

Cervical cancer is the second most prevalent cancer among women in developing nations, accounting for approximately 85% of global diagnoses and 88% of associated deaths in these regions [1]. While the incidence and mortality rates of cervical cancer have been decreasing due to effective diagnosis and vaccines, the global mortality rate of cervical cancer remains high at about 54% [2]. More than half of newly diagnosed cervical cancer patients are in the locally advanced stage, and platinum-based concurrent chemoradiotherapy (CCRT) has been utilized as a primary treatment for locally advanced cervical cancer (LACC) [3]. Advancements in radiotherapeutic techniques have partially improved the prognosis of patients with LACC. However, a notable portion of these cases still suffer from disease recurrence and distant metastasis [4]. Therefore, it is crucial to use more precise prognostic indicators based on preoperative clinical parameters to categorize high-risk patients with LACC to help optimize treatment strategies.

Inflammation plays a crucial role in tumorigenesis, immune responses, and treatment efficacy [5, 6]. Previous studies have demonstrated that several systemic inflammatory indicators, including neutrophil-to-lymphocyte ratio (NLR), lymphocyte-to-monocyte ratio (LMR), and platelet-to-lymphocyte ratio (PLR), are closely associated with poor survival outcomes in cervical carcinoma [7]. Additionally, nutritional status is another important prognostic factor for patients with cancer. Several studies have confirmed the correlation between nutritional deficiencies and elevated post-treatment complication rates, protracted hospitalizations, and poor survival [8, 9]. Wang et al. [10] found that cervical cancer patients with a lower prognostic nutritional index (PNI) and a lower geriatric nutritional risk index (GNRI) have shorter survival times. Recently, a novel biomarker, the Naples Prognostic Score (NPS), which combines inflammatory and nutritional elements, was proposed by Galizia et al. and showed favorable prognostic value in colorectal cancer [11], cholangiocarcinoma [12], lung cancer [13], and esophageal cancer [14]. However, there is currently a lack of studies on the prognostic value of NPS in LACC patients undergoing CCRT.

The aim of this study was to estimate the prognostic value of the NPS in patients with LACC undergoing CCRT. We aimed to investigate whether NPS could function as a predictive factor for survival outcomes and the occurrence of metastasis. We also compared the predictive efficacy of NPS with other indicators. Additionally, an NPS-based nomogram incorporating other clinical factors was established and validated.

## Materials and methods

### Patients selection

**Figure 1.**
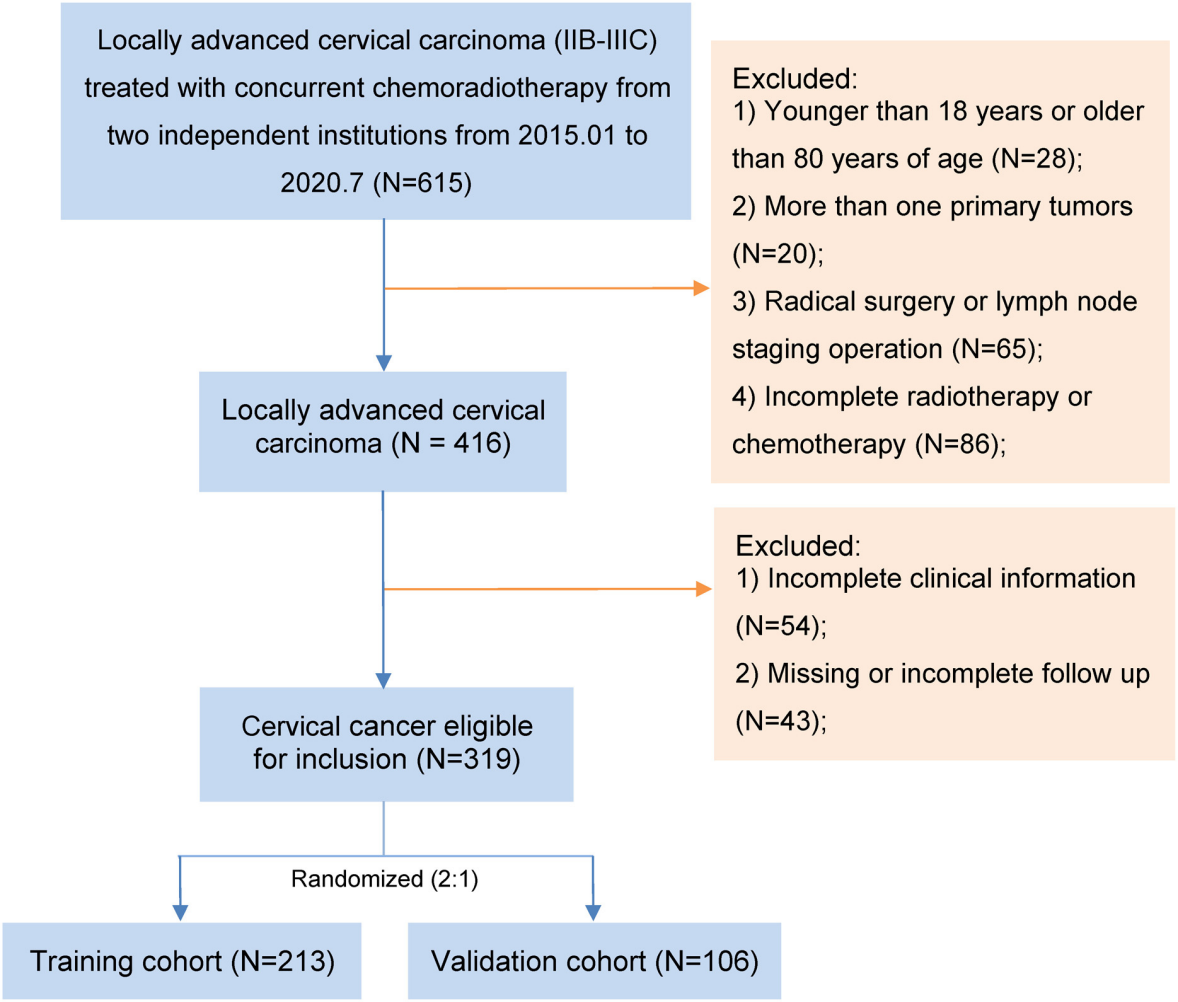
Flowchart of patient inclusion and exclusion.

A total of 319 LACC patients receiving CCRT at the Affiliated Hospital of Jiangnan University and the Affiliated Suzhou Hospital of Nanjing Medical University between January 2015 and July 2020 were retrospectively investigated. These patients were then randomly divided into a training set (*N* ═ 213) and a validation set (*N* ═ 106) in a 2:1 ratio. The following case selection criteria were defined: (1) clinical stage IIB–IIIC; (2) age 18–80 years; (3) adenocarcinoma or squamous cell carcinoma; (4) cervical cancer as the only diagnosed cancer; and (5) all patients received platinum-based CCRT plus high-dose-rate intracavitary brachytherapy. The exclusion criteria were as follows: (1) special pathological types, such as neuroendocrine cancer, sarcoma, and metastatic cancer; (2) patients undergoing radical surgery or lymph node staging operations; and (3) missing data for analysis. Figure 1 shows the flowchart of patient selection. This investigation was approved by the institutional review board of the Affiliated Hospital of iangnan University and the Affiliated Suzhou Hospital of Nanjing Medical University. All participants provided informed consent.

### Treatment and follow-up

The pelvic external irradiation dose was 45–50 Gy; with a fraction dose of 1.8–2.0 Gy per fraction, administrated once daily, five times a week. For patients with parametrial metastasis, an additional 5–10 Gy was administered; for patients with lymph node metastasis, an additional 10–20 Gy was given to the lymph nodes. Patients with para-aortic lymph node (PALN) metastasis received extended-field irradiation. Intracavitary radiotherapy was performed using two-dimensional high-dose-rate brachytherapy, 1–2 times per week, with each session delivering 6–7 Gy, completed in 4–5 fractions, with a total dose of 85–90 Gy to point A. Patients received concurrent platinum-based chemotherapy at a dose of 40 mg/m^2^ every week or a dose of 75 mg/m^2^ every three weeks.

Tumors were staged based on the 2018 International Federation of Gynecology and Obstetrics (FIGO) staging system. The survival outcomes assessed in this study include overall survival (OS) and progression-free survival (PFS). OS represents the duration between the initial diagnosis and death or the last visit, while PFS represents the interval from the initial diagnosis of LACC to the first occurrence of recurrence or cancer-related death. All patients adhered to regular systemic examinations: quarterly for the initial two years, semi-annually for the subsequent three years, and annually thereafter. The last follow-up was completed in July 2023.

### Definition of inflammatory and nutritional indicators

Hematological parameters were extracted from the clinical data repository of the hospitals within the 2-week period prior to the initiation of CCRT. The absolute counts of neutrophils, monocytes, platelets, lymphocytes, and serum albumin concentration were utilized to derive additional parameters, such as PLR, NLR, LMR, the systemic index of inflammation (SII), and PNI. The NPS was calculated using ALB, total blood cholesterol (TC), NLR, and LMR [11]. X-tile was used to define the optimal cutoffs of SII and PNI according to OS [15]. Detailed information on the calculation formulas for NPS, PLR, NLR, LMR, PNI, and SII can be found in Tables S1 and S2.

### Nomogram construction for OS and PFS and validation

Univariate and multivariate Cox regression analyses were conducted for OS and PFS, utilizing the proportional hazard hypothesis test to evaluate prognostic factors. Factors with *P* values less than 0.05 in the univariate Cox analysis were incorporated into the multivariate Cox regression model to identify independent prognostic indicators. Following the outcomes of the multivariate Cox analysis in the training cohort, nomograms were established. These nomograms integrated all the independent prognostic factors and were used as predictive tools for estimating the risk of patients surviving less than 12, 36, or 60 months. Each variable in the nomogram was assigned a specific number of points along a horizontal axis. By adding up the points corresponding to each patient’s variables, a risk score for that patient could be determined. The nomogram also provided estimates for the 12-, 36-, or 60-month OS and PFS rates based on the calculated risk score. The predictive ability of the nomogram model for OS and PFS in the training and validation cohorts was assessed using the C-index, time-dependent area under the curve (AUC), calibration curves, and decision-curve analysis (DCA).

### Ethical statement

This investigation was approved by the institutional review board of the Affiliated Hospital of iangnan University and the Affiliated Suzhou Hospital of Nanjing Medical University. All participants provided informed consent. This study was consistent with the Declaration of Helsinki.

### Statistical analysis

Categorical variables were analyzed using either chi-squared or Fisher’s exact tests, while *t*-tests were employed for continuous variables. The performance of the NPS was assessed against other inflammation-based indicators using receiver operating characteristic curve (ROC), with AUC comparisons providing a measure of discriminatory ability. The Kaplan–Meier method estimated OS and PFS rates, with log-rank tests determining their differences. Both univariate and multivariate analyses utilized a Cox proportional hazards regression model to explore the relationship between OS, PFS, and prognostic factors. Factors with a *P* < 0.05 in univariate analyses were incorporated into multivariable models. Statistical significance was set at a two-sided *P* < 0.05. All statistical analyses were conducted with R software version 4.2.1.

## Results

### Patient characteristics

Table 1 displays the baseline characteristics of the patients included in this study. In the training cohort, there were 213 LACC patients, with an average age of 56.75 ± 10.62 years. A total of 195 (91.5%) patients had squamous cell carcinoma, and 18 (8.5%) had adenocarcinoma. Based on the 2018 FIGO staging system, 66 individuals had lymph node metastases and were restaged to stage IIIC. Among them, 48 had pelvic lymph node (PLN) metastasis only, and 18 had both PLN and PALN metastasis. A total of 126 (59.2%) patients had a human papillomavirus (HPV) infection. Adjuvant chemotherapy (ACT) was administered to 179 (84%). The mean values for NLR, LMR, PLR, SII, and PNI were 3.04 ± 1.44, 4.41 ± 1.27, 156.89 ± 78.28, 702.88 ± 205.41, and 48.44 ± 5.90.

**Table 1:** Basic clinicopathologic characteristics of locally advanced cervical carcinoma in the training and validation cohort

**Characteristics**	**Training cohort**	**Validation cohort**	***P* value**
	***N* ═ 213**	***N* ═ 106**	
Age, mean ± SD	56.75 ± 10.62	57.34 ± 5.55	0.431
Histology, *N* (%)			0.476
SCC	196 (92.1%)	95 (89.6%)	
AC	17 (7.9%)	11 (10.4%)	
Differentiati on, *N* (%)			0.841
Well	21 (9.9%)	11 (10.4%)	
Moderate	157 (73.7%)	75 (70.8%)	
Poor	35 (16.4%)	20 (18.9%)	
FIGO, *N* (%)			0.392
IIB	85 (39.9%)	36 (34%)	
IIIA	21 (9.9%)	7 (6.6%)	
IIIB	41 (19.2%)	27 (25.5%)	
IIIC	66 (31.0%)	36 (34%)	
Tumor size, *N* (%)			0.712
≤ 4 cm	46 (21.6%)	21 (19.8%)	
> 4 cm	167 (78.4%)	85 (80.2%)	
Lymph node metastasis, *N* (%)			0.556
Negative	147 (69%)	70 (66%)	
PLN only	48 (22.5%)	23 (21.7%)	
PLN and PALN	18 (8.5%)	13 (12.3%)	
HPV infection, *N* (%)			0.112
Negative	126 (59.2%)	52 (49.1%)	
Positive	87 (40.8%)	54 (50.9%)	
ACT, *N* (%)			0.289
Yes	179 (84%)	84 (79.2%)	
No	34 (16%)	22 (20.8%)	
NLR, Mean ± SD	3.04 ± 1.44	3.08 ± 0.99	0.699
LMR, Mean ± SD	4.41 ± 1.27	4.58 ± 1.14	0.245
PLR, Mean ± SD	156.89 ± 78.28	148.17 ± 64.47	0.323
SII, Mean ± SD	702.88 ± 205.41	625.21 ± 201.11	0.168
PNI, Mean ± SD	48.44 ± 5.90	49.77 ± 6.09	0.644
NPS, *N* (%)			0.451
1	48 (22.5%)	29 (27.4%)	
2	100 (46.9%)	51 (48.1%)	
3	65 (30.5%)	26 (24.5%)	

AC: Adenocarcinoma; ACT: Adjuvant chemotherapy; FIGO: Federation of Gynecology and Obstetrics; HPV: Human papillomavirus; LMR: Lymphocyte to monocyte ratio; N: Number; NLR: Neutrophil to lymphocyte ratio; NPS: Naples Prognostic Score; PLAN: Para-aortic lymph node; PLN: Pelvic lymph node; PLR: Platelet-to-lymphocyte ratio; PNI: Prognostic nutritional index; SCC: Squamous cell carcinoma; SD: Standard deviation; SII: Index of systemic immune-inflammation.

In the validation cohort, there were 106 LACC patients with a average age of 57.34± 5.55 years. A total of 95 (89.6%) patients had squamous cell carcinoma, and 11 (10.4%) had adenocarcinoma. 36 cases had lymph node metastases and were restaged to stage IIIC. Among them, 23 had PLN metastasis only and 13 had both PLN and PALN metastasis. A total of 54 (50.9%) patients had an HPV infection. ACT was administered to 179 (84%). The mean values for NLR, LMR, PLR, SII, and PNI were 3.08 ± 0.99, 4.58 ± 1.14, 148.17 ± 64.47, 625.21 ± 201.11, and 49.77 ± 6.09.

### Prognostic comparison between NPS and other inflammation and nutrition indicators in the training set

**Figure 2.**
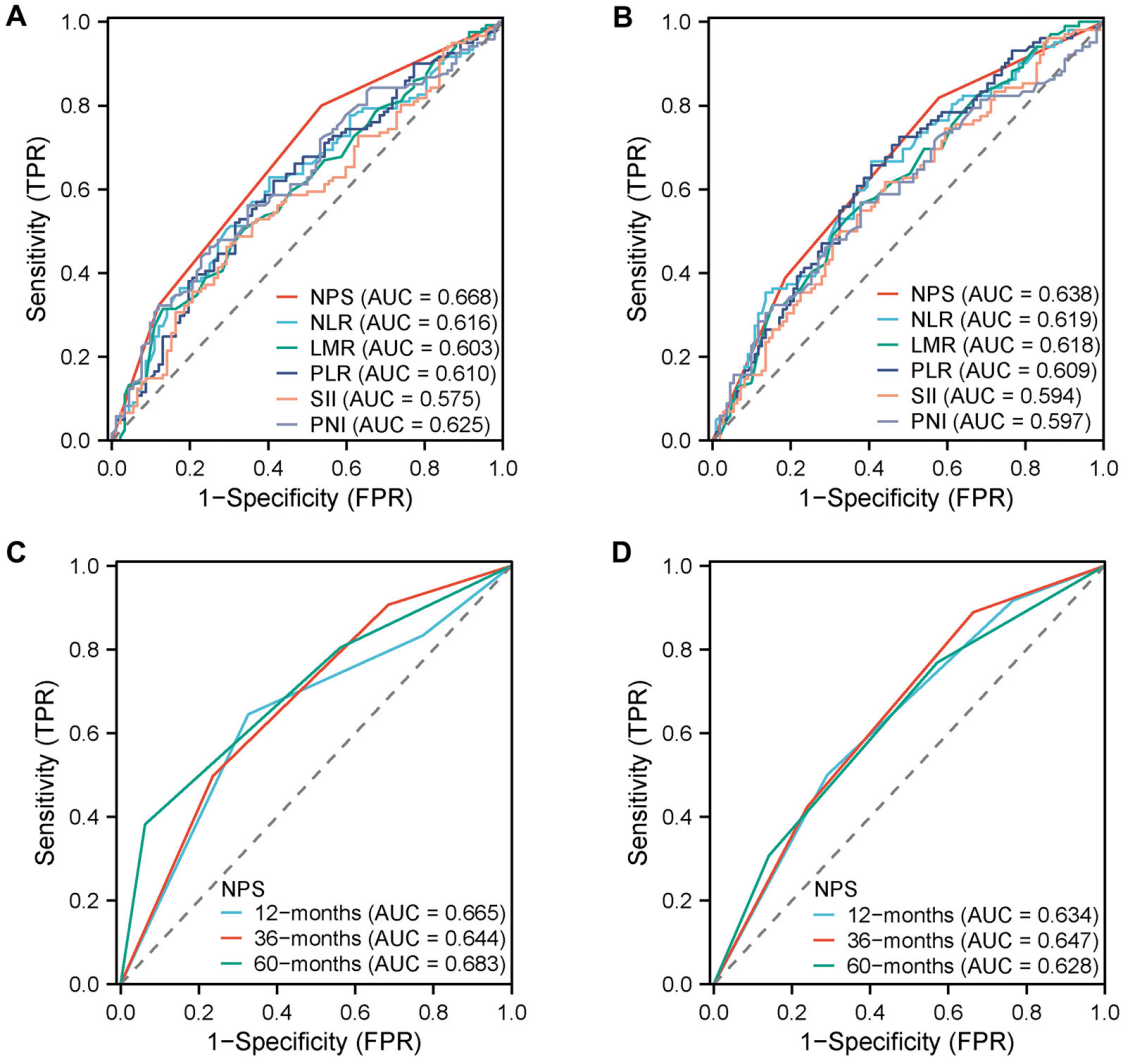
**AUC comparisons between NPS and other inflammation and nutrition indicators by ROC for OS (A) and PFS (B) in the training set.** The prognostic value of NPS in time-dependent ROC for OS (C) and PFS (D) in the training set. AUC: Area under the curve; LMR: Lymphocyte-to-monocyte ratio; NLR: Neutrophil-to-lymphocyte ratio; NPS: Naples Prognostic Score; PLR: Platelet-to-lymphocyte ratio; OS: Overall survival; PFS: Progression-free survival; PNI: Prognostic nutritional index; ROC: Receiver operating characteristic curve; SII: Systemic immune-inflammation index.

ROC analyses were conducted to evaluate the prognostic accuracy between NPS and other inflammation and nutrition indicators, such as NLR, LMR, PLR, SII, and PNI. NPS was found to have the largest AUC and better prognostic ability than other inflammation and nutrition indicators for predicting OS (AUC ═ 0.668) and PFS (AUC ═ 0.628) (Figure 2A and 2B). The time-dependent ROC curve is shown in Figure 2C and 2D.

### Associations between NPS and clinicopathological variables in the training set

In the training group, there were 48 (22.5%) cases in the NPS ═ 1 group, 100 (46.9%) cases in the NPS ═ 2 group, and the NPS ═ 3 group comprised 65 (30.5%) patients. Table 2 depicts the basic characteristics of individuals in different NPS groups. Based on the X-tile binary classification, 804.49 and 49.56 were defined as the optimal cutoff values for SII and PNI, respectively. NPS was found to have close relationships with FIGO stage (*P* < 0.045), HPV infection status (*P* ═ 0.016), SII (*P* < 0.001), and PNI (*P* < 0.001). No significant associations were found between NPS and age, histology, differentiation, tumor size, lymph node metastasis status, or ACT administration.

**Table 2:** Comparison of baseline clinicopathologic characteristics of locally advanced cervical carcinoma in the training cohort

**Characteristics**	**NPS ═ 1**	**NPS ═ 2**	**NPS ═ 3**	***P* value**
	**(*N* ═ 48)**	**(*N* ═ 100)**	**(*N* ═ 65)**	
Age, mean ± SD	54.35 ± 9.95	55.77 ± 10.29	53.47 ± 11.56	0.384
Age, *N* (%)				0.568
≤ 60 years	33 (68.8%)	60 (60.0%)	42 (64.6%)	
> 60 years	15 (31.2%)	40 (40.0%)	23 (35.4%)	
Histology, *N* (%)				0.541
SCC	44 (91.7%)	94 (94.0%)	58 (89.2%)	
AC	4 (8.3%)	6 (6.0%)	7 (10.8%)	
Differentiation, *N* (%)				0.775
Well	7 (14.6%)	9 (9.0%)	5 (7.7%)	
Moderate	33 (68.8%)	74 (74.0%)	50 (76.9%)	
Poor	8 (16.7%)	17 (17.0%)	10 (15.4%)	
FIGO, *N* (%)				0.045
IIB	23 (47.9%)	43 (43.0%)	19 (29.2%)	
III	25 (52.1%)	57 (57.0%)	47 (70.8%)	
Tumor size, *N* (%)				0.941
≤ 4 cm	10 (20.8%)	21 (21.0%)	15 (23.1%)	
> 4 cm	38 (79.2%)	79 (79.0%)	50 (76.9%)	
Lymph node metastasis, *N* (%)				0.357
Negative	30 (62.5%)	72 (72.0%)	45 (69.2%)	
PLN only	11 (22.9%)	20 (20.0%)	17 (26.2%)	
PLN and PALN	7 (14.6%)	8 (8.0%)	3 (4.6%)	
HPV infection, *N* (%)				0.016
Negative	32 (66.7%)	65 (65.0%)	29 (49.2%)	
Positive	16 (33.3%)	35 (35.0%)	36 (50.8%)	
ACT, *N* (%)				0.605
Yes	39 (81.2%)	83 (83.0%)	57 (87.7%)	
No	9 (18.8%)	17 (17.0%)	8 (12.3%)	
SII				< 0.001
≤ 804.49	30 (62.5%)	32 (32.0%)	13 (20.0%)	
> 804.49	18 (37.5%)	68 (68.0%)	52 (80.0%)	
PNI				< 0.001
≤ 49.56	9 (18.8%)	42 (42.0%)	47 (72.3%)	
> 49.56	39 (81.2%)	58 (58.0%)	18 (27.7%)	

AC: Adenocarcinoma; ACT: Adjuvant chemotherapy; FIGO: The Federation of Gynecology and Obstetrics; HPV: Human papillomavirus; N: Number; NPS: Naples Prognostic Score; PLAN: Para-aortic lymph node; PLN: Pelvic lymph node; PNI: Prognostic nutritional index; SCC: Squamous cell carcinoma; SD: Standard deviation; SII: Index of systemic immune-inflammation.

### Survival analysis in the training set

**Figure 3.**
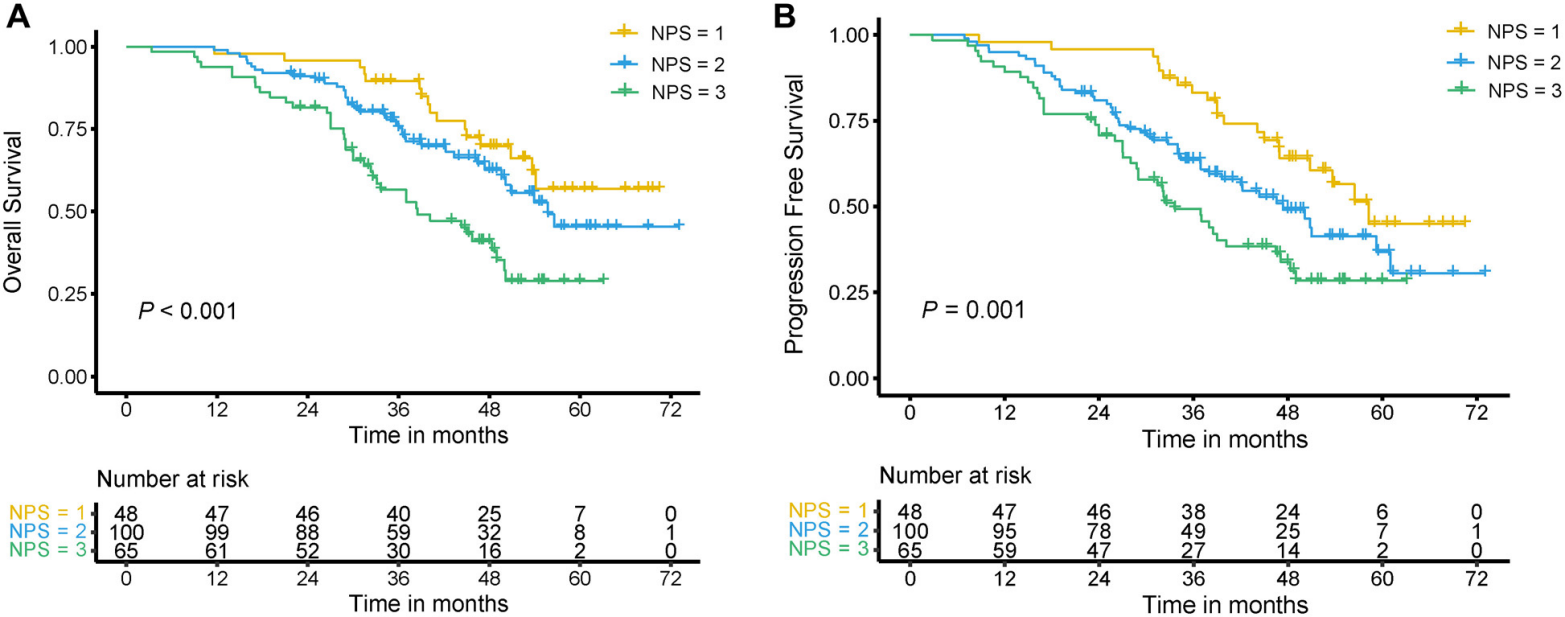
**Survival differences for OS (A) and PFS (B) among different NPS groups in locally advanced cervical cancer patients treated with chemoradiotherapy in the training set.** NPS: Naples Prognostic Score; OS: Overall survival; PFS: Progression-free survival.

The 5-year OS for the NPS ═ 1, 2, and 3 groups was 56.8%, 45.4%, and 28.9% (*P* < 0.001), and the 5-year PFS for the NPS ═ 1, 2, and 3 groups was 44.9%, 36.7%, and 28.4% (*P* ═ 0.001), respectively. The Kaplan–Meier curves of OS and PFS are shown in Figure 3A and 3B.

### Cox regression analysis for prognostic factors in the training set

Univariate Cox analyses for OS showed that histology (*P* ═ 0.014), FIGO (*P* ═ 0.016), lymph node metastasis (PLN only vs negative, *P* ═ 0.790; PLN and PALN vs negative, *P* ═ 0.048), SII (*P* ═ 0.039), PNI (*P* ═ 0.021), and NPS (NPS ═ 2 group vs NPS ═ 1 group, *P* ═ 0.046; NPS ═ 3 group vs NPS ═ 1 group, *P* < 0.001) were important predictors for OS. Histology (*P* ═ 0.042), FIGO (*P* ═ 0.036), lymph node metastasis (PLN only vs negative, *P* ═ 0.047; PLN and PALN vs. negative, *P* ═ 0.035), HPV infection (*P* ═ 0.044), SII (*P* ═ 0.045), PNI (*P* ═ 0.041), and NPS (NPS ═ 2 group vs NPS ═ 1 group, *P* ═ 0.049; NPS ═ 3 group vs NPS ═ 1 group, *P* < 0.001) were important predictors for PFS.

Multivariate Cox analyses demonstrated that histology (hazard ratio [HR] ═ 1.676, *P* ═ 0.044), FIGO (HR ═ 1.810, *P* ═ 0.016), lymph node metastasis (PLN only vs negative, HR ═ 1.095, *P* ═ 0.512; PLN and PALN vs negative, HR ═ 1.922, *P* ═ 0.023), SII (HR ═ 1.298, *P* ═ 0.044), PNI (HR ═ 1.661, *P* ═ 0.019), and NPS (NPS ═ 2 group vs NPS ═ 1 group, HR ═ 1.334, *P* ═ 0.041; NPS ═ 3 group vs NPS ═ 1 group, HR ═ 2.799, *P* < 0.001) could serve as independent predictors for OS. Histology (HR ═ 1.572, *P* ═ 0.048), FIGO (HR ═ 1.544, *P* ═ 0.041), lymph node metastasis (PLN only vs negative, HR ═ 1.803, *P* ═ 0.041; PLN and PALN vs negative, HR ═ 1.352, *P* ═ 0.032), HPV infection (HR ═ 0.606, *P* ═ 0.011), SII (HR ═ 1.134, *P* ═ 0.045), PNI (HR ═ 1.457, *P* ═ 0.022), and NPS (NPS ═ 2 group vs NPS ═ 1 group, HR ═ 1.765, *P* ═ 0.037; NPS ═ 3 group vs NPS ═ 1 group, HR ═ 2.738, *P* < 0.001) could serve as independent predictors for PFS. Table 3 exhibits the univariate and multivariate Cox analyses outcomes.

**Table 3:** Univariable and multivariable cox analyses associated with OS in the training cohort

**Characteristics**	** OS Univariate analysis**		**OS Multivariate analysis**		**PFS Univariate analysis**		**PFS Multivariate analysis**	
	**Hazard ratio (95% CI)**	***P* value**	**Hazard ratio (95% CI)**	***P* value**	**Hazard ratio (95% CI)**	***P* value**	**Hazard ratio (95% CI)**	***P* value**
*Age*								
≤ 60 years	Reference				Reference			
> 60 years	0.886 (0.575–1.365)	0.583			0.976 (0.662–1.438)	0.902		
*Histology*								
SCC	Reference		Reference		Reference		Reference	
AC	2.088 (1.161–3.755)	0.014	1.676 (1.024–3.041)	0.044	1.826 (1.022–3.262)	0.042	1.572 (1.008–2.921)	0.048
*Differentiation*								
Well	Reference				Reference			
Moderate	1.397 (0.640–3.048)	0.401			1.342 (0.673–2.677)	0.403		
Poor	2.262 (0.955–5.356)	0.063			1.851 (0.851–4.023)	0.120		
*FIGO*								
IIB	Reference		Reference		Reference		Reference	
III	1.880 (1.123–3.148)	0.016	1.810 (1.078–3.038)	0.025	1.601 (1.032–2.486)	0.036	1.544 (1.089–2.401)	0.041
*Tumor size*								
≤ 4 cm	Reference				Reference			
> 4 cm	0.793 (0.490–1.283)	0.344			0.784 (0.503–1.220)	0.281		
*Lymph node metastasis*								
Negative	Reference		Reference		Reference		Reference	
PLN only	1.073 (0.640–1.798)	0.790	1.095 (0.621–1.354)	0.512	1.788 (1.010–3.175)	0.047	1.803 (1.012–3.132)	0.041
PLN and PALN	1.831 (1.009–3.337)	0.048	1.922 (1.149–3.568)	0.023	1.558 (1.267–2.278)	0.035	1.352 (1.271–2.288)	0.032
*HPV infection*								
Positive	Reference				Reference		Reference	
Negative	0.713 (0.473–1.076)	0.107			0.680 (0.467–0.990)	0.044	0.606 (0.412–0.889)	0.011
*ACT*								
Yes	Reference				Reference			
No	0.837 (0.465–1.505)	0.552			0.822 (0.476–1.418)	0.481		
*SII*								
≤ 804.49	Reference		Reference		Reference		Reference	
> 804.49	1.944 (1.103–2.580)	0.039	1.298 (1.013–2.680)	0.044	1.125 (1.012–1.332)	0.045	1.134 (1.014–1.358)	0.045
*PNI*								
> 49.56	Reference		Reference		Reference		Reference	
≤ 49.56	1.632 (1.078–2.472)	0.021	1.661 (1.087–2.537)	0.019	1.432 (1.073–2.109)	0.041	1.457 (1.181–2.572)	0.022
*NPS*								
1	Reference		Reference		Reference		Reference	
2	1.461 (1.012–2.629)	0.046	1.334 (1.138–2.414)	0.041	1.687 (1.002–2.843)	0.049	1.765 (1.036–3.008)	0.037
3	2.897 (1.613–5.203)	<0.001	2.799 (1.558–5.029)	<0.001	2.595 (1.518–4.436)	<0.001	2.738 (1.599–4.690)	<0.001

AC: Adenocarcinoma; ACT: Adjuvant chemotherapy; FIGO: Federation of Gynecology and Obstetrics; HPV: Human papillomavirus; N: Number; NPS: Naples Prognostic Score; OS: Overall survival; PFS: Progression-free survival; PLAN: Para-aortic lymph node; PLN: Pelvic lymph node; PNI: Prognostic nutritional index; SCC: Squamous cell carcinoma; SII: Index of systemic immune-inflammation.

### Nomogram establishment and validation

**Figure 4.**
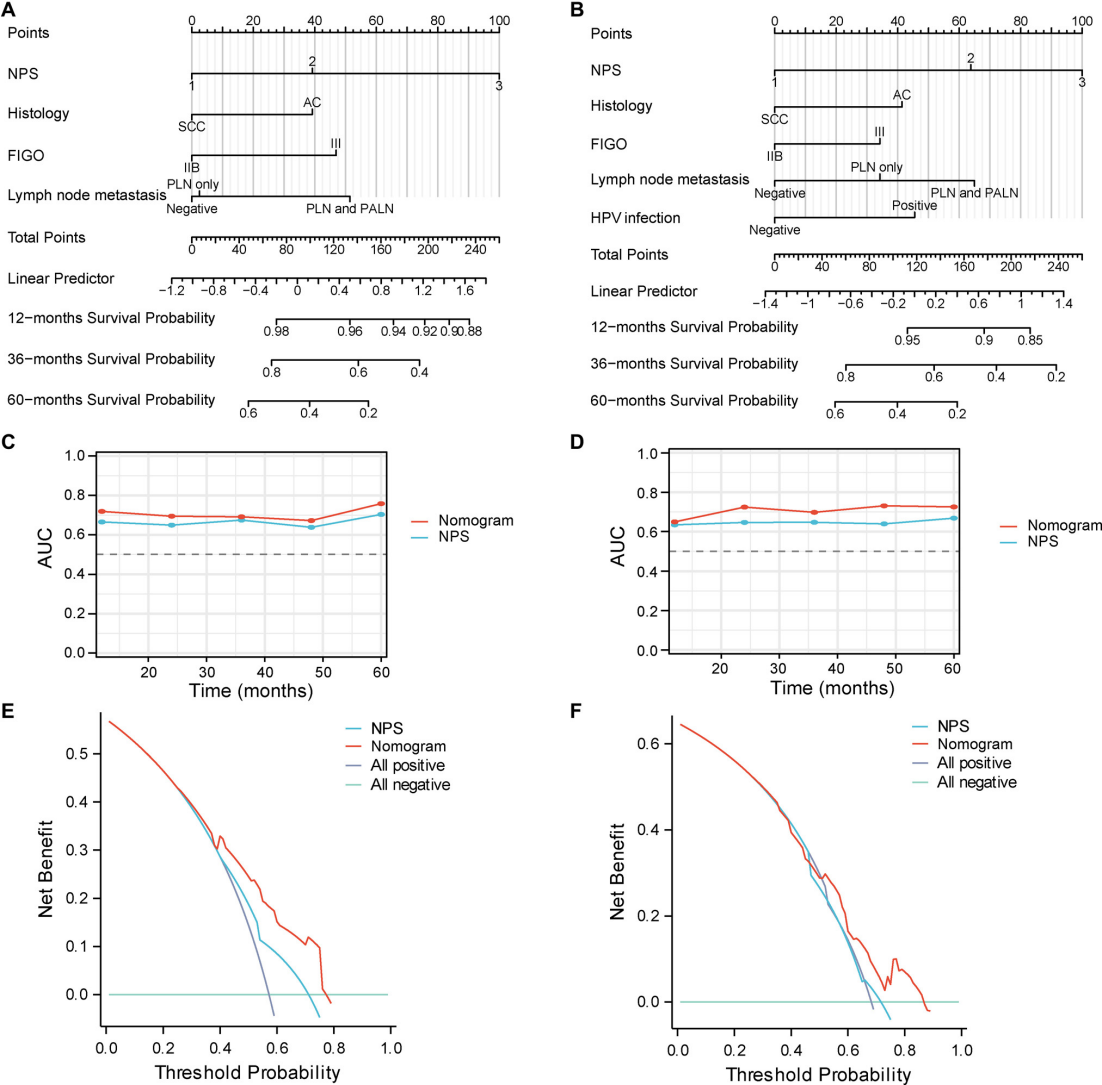
**Nomograms to predict OS (A) and PFS (B) for patients with locally advanced cervical cancer in the training set.** Time-dependent AUC values for the nomograms and NPS for OS (C) and PFS (D) in the training set. The decision-curve analysis plots of the nomograms for 60-month OS (E) and PFS (F) in the training set. AUC: Area under the curve; FIGO: The International Federation of Gynecology and Obstetrics; HPV: Human papillomavirus; NPS: Naples Prognostic Score; OS: Overall survival; PFS: Progression-free survival; PALN: Para-aortic lymph node; PLN: Pelvic lymph node.

**Figure 5.**
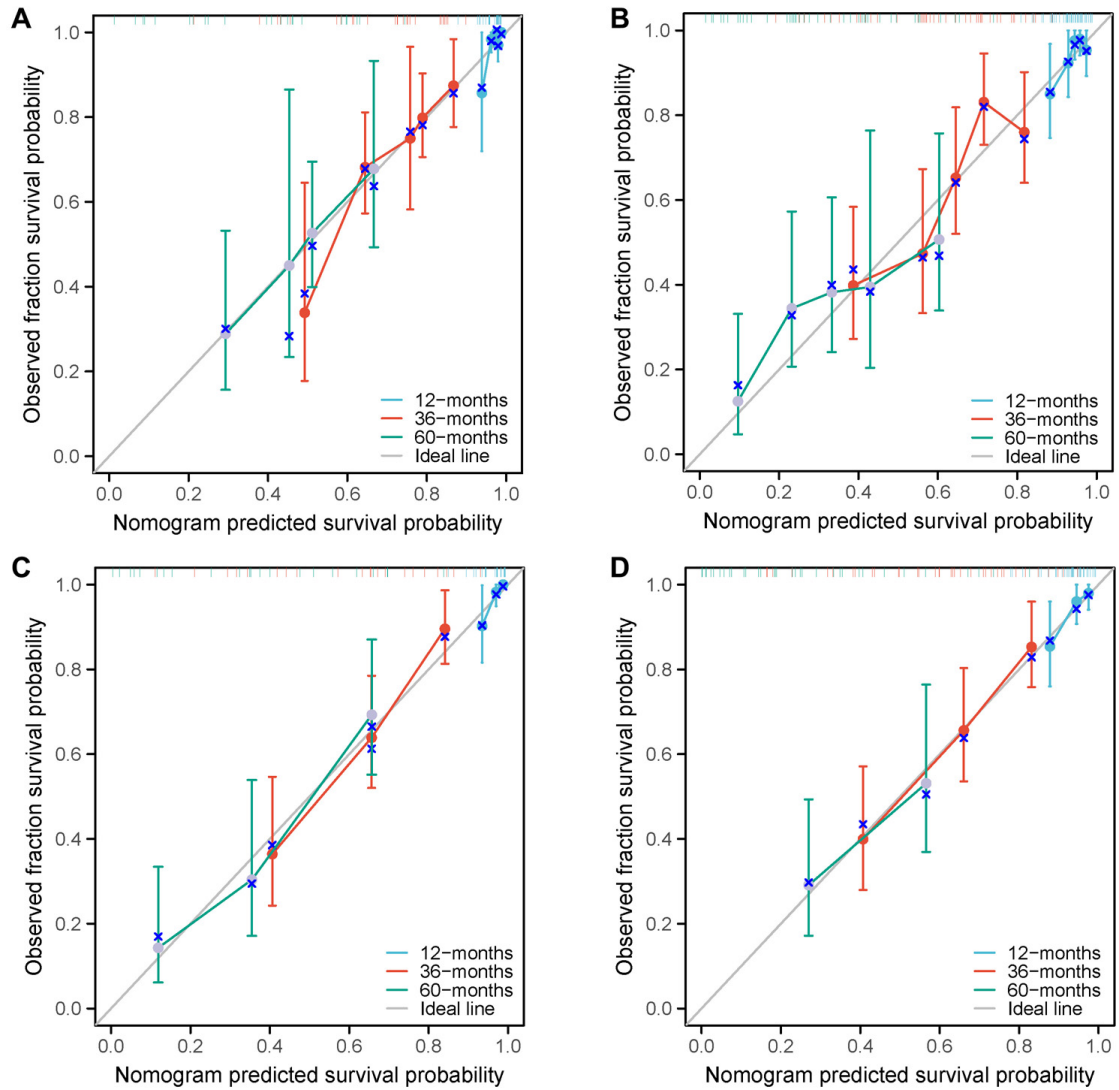
Calibration curves of the nomograms for 12-, 36-, and 60-month overall survival and progression-free survival in the training set (A and B) and validation set (C and D).

All the independent factors identified in the prognostic analysis for OS and PFS were incorporated into the predictive models and then visually presented as nomograms (Figure 4A and 4B). To avoid the repeated inclusion of inflammation and nutrition indicators, only NPS was included for nomogram construction. The risk scores for each of the 213 patients in the training set and validation set were obtained using the nomograms. The C-indexes of the nomogram in the training set were 0.722 for OS and 0.683 for PFS, while in the validation set, the C-indexes were 0.731 for OS and 0.693 for PFS. Time-dependent AUC values of risk scores and NPS for OS and PFS are displayed in Figure 4C and 4D. The DCA plots for the 60-month rates of OS and PFS in both the training set and validation set are exhibited in Figure 4E and 4F. The calibration curves indicated a high level of concordance between the observed events and the predicted probabilities for both OS and PFS at 12, 36, and 60 months in the training set (Figure 5A and 5B) and validation set (Figure 5C and 5D).

## Discussion

This study first estimated the relationship between NPS and prognosis in LACC patients undergoing CCRT. Patients with higher NPS had worse survival outcomes. The predictive efficacy of NPS was compared with other inflammation and nutrition markers, such as NLR, LMR, PLR, SII, and PNI. We found that NPS had better predictive ability for OS and PFS than these common inflammation and nutrition indicators and served as an independent prognostic factor. Hence, NPS could be a potential biomarker for identifying LACC patients with inferior long-term prognoses. Additionally, we developed new prognostic nomograms based on NPS and other independent prognostic factors to enhance the prediction of OS and PFS in LACC patients. A favorable identification was observed in the validation set.

Previous studies have summarized that several postoperative pathologic indicators have a close association with the prognosis of LACC, including lympho-vascular space invasion (LVSI), perineural invasion, depth of stromal invasion (DOI), and tumor-infiltrating lymphocytes (TILs) [16]. Several studies have explored the prognostic significance of LVSI concerning nodal and distant metastases, as well as patient survival. However, the findings are highly heterogeneous. Some studies indicate that LVSI has a negative prognostic impact, while others have not demonstrated statistically significant associations [17–19]. The prognostic significance of perineural invasion is limited; however, it is widely recognized that perineural invasion is often associated with other adverse prognostic factors, including LVSI, deep stromal invasion, larger tumor size, and parametrial invasion [20]. A recent meta-analysis indicated that a cutoff of 3 mm for residual stromal invasion appears to be superior to other residual tumor scoring systems in terms of prognostic stratification for cervical cancer patients after neoadjuvant treatment [21]. A higher prevalence of CD4+ and CD8+ T cells is observed to have a close association with improved patient outcomes [22]. As such, a precise characterization of TILs may offer valuable insights not only for patient prognosis but also for guiding therapeutic management strategies. However, obtaining these postoperative pathological indicators necessitates biopsy or surgical resection of the primary lesion. In contrast, predicting patient prognosis using blood-based markers may offer a more convenient approach in clinical practice.

Inflammation significantly influences tumor development and progression, as indicated by hematological parameters [23]. Increased neutrophil and monocyte concentrations in the tumor environment can suppress immunity and promote tumor growth by triggering myeloid-derived suppressor cells [24, 25]. The interactions between tumor cells and platelets can induce platelet activation and aggregation, leading to the promotion of cancer-associated thrombosis and contributing to tumor metastasis [26]. Conversely, lymphocytes are vital in restraining tumor cell proliferation and metastasis [27]. Several studies have investigated the association between these peripheral blood parameter ratios and the prognosis of cervical carcinoma. A recent meta-analysis evaluated the prognostic value of several systemic hemato-immunological indicators for the treatment of cervical cancer [7]. This meta-analysis incorporated 22 retrospective cohort studies, totaling 9558 patients. The results revealed a significant correlation between elevated NLR, PLR, TLR, and CAR levels and a poor prognosis in cervical cancer patients. Subgroup analysis further indicated that NLR and PLR functioned as more precise biomarkers in FIGO stage I-III cervical cancer patients treated with chemoradiotherapy. Notably, high LMR exhibited significant prognostic implications for late-stage (FIGO III–IV) cervical cancer. Another study, the ESTHER study, also analyzed the correlation between systemic inflammation indices and therapeutic outcomes in LACC patients undergoing definitive chemoradiation [28]. A total of 173 cases were enrolled, and the prognostic abilities of NLR, PLR, MLR, SII, combination of PLT and NLR (COP-NLR), and the systemic inflammatory response index (SIRI) for OS and disease-free survival (DFS) were compared. Only SII was found to have a significant association with DFS but not with OS in this study. In contrast to other studies showing a significant correlation between pretreatment inflammation indicators and DFS [29–31] and OS [30, 32, 33], this study attributes the discrepancy to the excessive presence of confounding factors and the lack of predefined cutoff values or cutoff values defined based on ROC curve analysis to assess the indicators, instead applying their values as continuous variables. Although the above meta-analysis did not observe a significant correlation between SII and OS in cervical cancer, the findings of our study align with those of Huang et al. and Guo et al., all demonstrating that high pretreatment SII was significantly associated with poor OS [23, 34].

Recently, the influence of nutritional status on the prognosis of cancer patients has garnered attention. Malnourished patients often have a lower tolerance to treatment, leading to more severe side effects and complications [35]. This can result in longer hospital stays and higher medical costs. Additionally, malnutrition weakens the immune system, leading to a poorer prognosis and survival rate. Several studies have confirmed that nutrition indicators are closely associated with survival outcomes in various cancers, including cervical cancer [36], breast cancer [37], colorectal cancer [38], bladder cancer [39], lung cancer [40], endometrial cancer [41], and esophageal cancer [42]. ALB, controlling nutritional status (CONUT), PNI, GNRI, and nutritional risk index (NRI) are common nutrition indicators that can be calculated using peripheral blood parameters. A study by Wang et al. [43] revealed that cervical cancer patients with hypoproteinemia have a worse prognosis compared to those without. Similarly, Tan et al. [44] discovered that CONUT could serve as an independent prognostic factor in cervical cancer patients, with those having a high preoperative CONUT score being correlated with poor OS and DFS. Another meta-analysis encompassing nine articles and 2508 cases showed that a low PNI foretells poor OS and PFS in cervical cancer patients [45].

Given that both inflammation and nutritional state have an impact on the cancer prognosis, Galizia et al. [11] first reported NPS, incorporating ALB, TC, NLR, and LMR, as a robust prognostic indicator in 2017 for colorectal patients treated with surgery. Then the favorable prognostic value of NPS or modified NPS was confirmed in other malignancies [46, 47]. Immunonutritional indicators have also been explored to predict survival outcomes in cervical cancer. Chen et al. [36] conducted a study where they combined the PNI and NLR to predict the toxicity and prognosis of cervical cancer patients undergoing chemoradiotherapy. Their results indicated that pretreatment immunonutritional metrics might function as quantitative indicators for forecasting survival rates and treatment-related toxicities in individuals with cervical cancer undergoing definitive chemoradiotherapy. However, applying multimodal prognostic indicators in cancer management is complex. We are diligently investigating the predictive potential of NPS in LACC patients. The ideal cutoff values for NLR, LMR, ALB, and TC, as per Galizia’s study, do not account for individual study variabilities. Nonetheless, this does not impact our conclusions.

We discovered a significant association between NPS and clinicopathological variables, specifically FIGO stage and HPV infection status. Similarly, Flores-Cisneros et al. [48] found that LACC patients in advanced stages tend to have poorer nutritional status prior to treatment. Previous studies have shown that HPV has the potential to elevate the levels of cyclooxygenase (COX)-2 and prostaglandin (PG) E2, triggering the COX-PG pathway in cervical cancer, which is considered the primary driver of HPV-induced inflammation [49]. Furthermore, HPV oncogenes can enhance the production of proinflammatory cytokines in HPV-positive individuals, accelerating inflammation after HPV infection. Results of multivariate analysis in our study demonstrated that NPS and FIGO stage were independent prognostic factors for OS and PFS, and HPV infection status was an independent risk factor for PFS.

A recent study demonstrated that the E5 and E6/E7 oncoproteins of high-risk HPV contribute to immune evasion by upregulating the programmed cell death-1/programmed cell death-ligand 1 (PD-1/PD-L1) pathway [50]. The study by Qin et al. demonstrated that HPV-induced somatic mutations significantly contribute to creating an inhibitory tumor microenvironment. This environment results in the aberrant expression of checkpoint-related genes, such as CTLA-4, PD-1, and PD-L1, which can promote immune evasion and tumor progression [51]. The E5 oncoprotein activates EGFR, which in turn enhances YAP activity, leading to the upregulation of PD-L1. This process initiates T cell apoptosis, contributes to persistent HPV infections, and increases the risk of cervical cancer development [52]. Studies suggest that the E6 and E7 oncoproteins activate the PD-1/PD-L1 axis, resulting in increased expression of Th2-type cytokines and IL-10, while reducing the expression of the Th1 cytokine IFN-γ and IL-12 [53, 54]. This shift contributes to immunosuppression and the further progression of cervical intraepithelial neoplasia (CIN). Interestingly, this study observed that the proportion of HPV infection is higher among LACC patients with high NPS. In HPV-infected cervical cancer patients, an increased proportion of neutrophils may correlate with a higher NLR, which could influence NPS values [55].

Our study was the first to investigate the relationship between pretreatment NPS and survival prognosis in LACC patients undergoing CCRT. However, there are several limitations. Firstly, the retrospective study design may introduce selection and information bias. Secondly, despite excluding patients with hematological disorders or those on immunomodulatory treatment, other conditions could potentially affect blood-based biomarkers. Thirdly, the limited sample size may restrict the application of our findings. Lastly, the cutoff values for NLR, LMR, Alb, and TC, determined using Galizia et al.’s method, may lack specificity.

## Conclusion

In conclusion, pretreatment NPS has a significant predictive value for OS and PFS in LACC patients undergoing CCRT. NPS could potentially guide personalized treatment for these patients. Nevertheless, further studies with larger sample sizes are warranted to validate these findings.

## Supplemental data

**Table S1:** Calculation of NPS

**Factor**	**Cut-off value**	**Points**	**NPS group**
Serum albumin (g/L)	≥ 40	0	NPS 1: 0 point NPS 2: 1 or 2 points NPS 3: 3 or 4 points
	< 40	1	
Total cholesterol (mg/dL)	> 180	0	
	≤ 180	1	
Neutrophils/lymphocytes	≤ 2.96	0	
	> 2.96	1	
Lymphocytes/monocytes	> 4.44	0	
	≤ 4.44	1	

NPS: Naples Prognostic Score.

**Table S2:** Inflammatory markers of interest

**Inflammatory marker**	**Laboratory parameters**	**Formula**
Platelet-to-lymphocyte ratio (PLR)	Platelets; Lymphocytes	Platelets/lymphocytes
Neutrophil-to-lymphocyte ratio (NLR)	Neutrophils; Lymphocytes	Neutrophils/lymphocytes
Lymphocyte-to-monocyte ratio (LMR)	Monocytes; Lymphocytes	Lymphocytes/monocytes
Systemic index of inflammation (SII)	Platelets; Neutrophils; Lymphocytes	Platelets × (Neutrophils/Lymphocytes)
Prognostic nutritional index (PNI)	Serum albumin; Lymphocyte count	ALB(g/L) + 0.005 × Lymphocytes

PLR: Platelet-to-lymphocyte ratio; NLR: Neutrophil to lymphocyte ratio; LMR: Lymphocyte to monocyte ratio; SII: Index of systemic immune inflammation; PNI: Prognostic nutritional index.

## Data Availability

The data that support the findings of this study are available from the corresponding author upon reasonable request.
